# Neutron Bragg-edge transmission imaging for microstructure and residual strain in induction hardened gears

**DOI:** 10.1038/s41598-021-83555-9

**Published:** 2021-02-18

**Authors:** Yuhua Su, Kenichi Oikawa, Takenao Shinohara, Tetsuya Kai, Takashi Horino, Osamu Idohara, Yoshitaka Misaka, Yo Tomota

**Affiliations:** 1 J-PARC Center, Japan Atomic Energy Agency, 2-4 Shirakata, Tokai, Ibaraki 319-1195 Japan; 2 Research and Development Headquarters, Neturen Co., Ltd., 7-4-10, Tamura, Hiratsuka-shi, Kanagawa 254-0013 Japan; 3 National Institute of Advanced Industrial Science and Technology, 1-1-1 Umezono, Tsukuba, Ibaraki 305-0047 Japan

**Keywords:** Engineering, Materials science

## Abstract

A time-of-flight Bragg-edge neutron transmission imaging was used to investigate the microstructure and strain distributions in a gear hardened by a newly developed two-step induction-heating method: precursor (Sample 1) and final product (Sample 2). The edge-position and edge-broadening were determined and mapped with high spatial resolution, which enabled us to confirm the two-dimensional distributions of the microstructure and residual strain. A deep hardened layer was made for Sample 1 in which martensite was formed on the entire teeth and the outer peripheral portion of the gear body. Sample 2 was subjected to double induction-hardening, where a tempered martensite was formed as the thermal refined microstructure between a fine-grained martensite at the tooth surface and a ferrite-pearlite microstructure at the core. The relationship between edge-broadening and the Vickers hardness described by a linear equation was employed to derive the elastic residual strain. The residual strain map for Sample 2 revealed that a steep compressive strain was introduced into the fine-grained martensite at the tooth surface by the super rapid induction-heating and quenching process. The reversal of tension was speculated to occur below 2 mm from the tooth tip, and the strain was almost zero in the core region.

## Introduction

In recent years, the demand for modern gear designs for products with reduced noise, weight, and size has increased. Furthermore, cost containment is also mostly required in the development process. High gear quality requires careful consideration of the gear size, gear geometry, materials, and a suitable heat treatment. Among the various heat-treating processes employed, the use of induction hardening has continually increased. Induction hardening, as an ecological and economical method, leads to significant improvement in the mechanical properties and enables the downsizing and weight reduction of the components. This process employs very rapid heating with clean electrical induction for a short period. The process is extremely energy efficient because only the part of the component that requires treatment is selectively heated and a fine-grained martensite microstructure is formed in the surface layers^1–3^. Moreover, the hardness values are strongly related to the evolution of the ferrite-martensite microstructure of steels, and the extreme hardness is due to the formation of the hard phase, martensite. Speich and Miller^4^ have reported that the hardness of a low carbon alloy ferrite-martensite dual phase (DP) steel is linearly dependent on the martensite volume fraction.

Residual stress and microstructure, which are significantly affected by heat treatment, play an important role in attaining the desired properties of gear products. For example, stress generation during induction hardening is very complex, owing to the selective heating induced by high temperature gradients in the sample^5,6^. Typically, the residual stress and microstructure in engineering materials are investigated via conventional techniques, such as electron backscatter diffraction (EBSD)^7^, X-ray diffraction (XRD)^8^, neutron diffraction^9,10^, and the finite element method (FEM)^11^. Bragg-edge imaging, which has been developed since around 2000, offers the possibility for non-destructive visualization of the microstructural characteristics, such as texture variations, crystalline phases, crystallite sizes, and lattice strains, of the sample with high spatial resolution^12–15^. Lehmann et al. used an energy-selective neutron imaging approach to extract volume microstructural information from welded steel and aluminum joints^16^. The selection of narrowed neutron wavelength bands was performed at the imaging facility ICON at the Swiss spallation neutron source (Paul Scherrer Institute) using a turbine-type device, which enables a 15% resolution in Δλ/λ. Moreover, Woracek et al. used neutron transmission to perform three-dimensional (3D) mapping of crystallographic phases in stainless steels subjected to tension or torsion. This mapping was achieved through wavelength selectivity at ~ Δλ/λ = 3% using a tunable double-crystal monochromator at the CONRAD beamline housed at the reactor source (Helmholtz-Zentrum Berlin, Germany). A spatial resolution of ≈ 100–300 μm was realized^17^. Furthermore, strain mapping is possible with residual strain deduced from the Bragg-edge position, especially for time-of-flight (TOF) Bragg-edge transmission imaging at short-pulsed neutron sources where high wavelength resolution of ~ 0.2–0.3% is possible. Santisteban et al. visualized the strain variations around a cold expanded hole in a steel plate for the first time ever by measuring the TOF Bragg-edge transmission spectra. Measurements were performed using a two-dimensional (2D) detector consisting of a 10 × 10 array of 2 × 2 mm^2^ at the ENGIN instrument (ISIS Facility, Rutherford Laboratory, UK)^18^. More recently, Tremsin et al. obtained strain maps with high spatial resolution (sub-millimeter level) using a 2D counting detector to perform in-situ TOF transmission imaging measurements on torqued steel bolts^19^. Recently, to address Bragg-edge broadening resulting from martensite phase formation in a quenched ferritic steel rod sample, Sato et al. revised the RITS (Rietveld Imaging of Transmission Spectra) code. This revision included deconvolution of the edge-profile function into the instrumental resolution function and the Gaussian distribution function^20^. The Bragg-edge imaging was performed at BL10 NOBORU (wavelength resolution: 0.33% in Δλ/λ) at the Materials and Life Sciences Experimental Facility (MLF) of the Japan Proton Accelerator Research Complex (J-PARC)^21^. As a result, the *d*-spacing, *d*_hkl_, and the full width at half maximum (FWHM) of a Gaussian *d*-spacing distribution, *w*_hkl_, i.e., broadening of the Bragg-edge for the hkl indices, related to microscopic-strain and crystallite size were simultaneously obtained from detailed analysis of a single Bragg-edge position and width. Additionally, they deduced that the *w*_hkl_ was linearly proportional to the Vickers hardness, Hv, which was linearly proportional to the quantity of ferrite/martensite. Their results suggested that the microhardness of a quenched ferritic steel product can be quantitatively mapped using this method.

A newly developed procedure, DIQ, a double induction quenching process, which is very effective for improving the fatigue strength of induction hardened gear products, has been used for the low-cost production of gears with improved stability and high precision^22,23^. In the present study, we applied the Bragg-edge broadening analysis method to this product. Using this technique, we analyzed the residual strain and microstructural distribution associated with two types of induction hardened gears. We also examined the correlation between the Vickers hardness obtained by means of conventional methods and the Bragg-edge broadening obtained from the transmission data analysis of the hardened gears.

## Experimental

**Figure 1:**
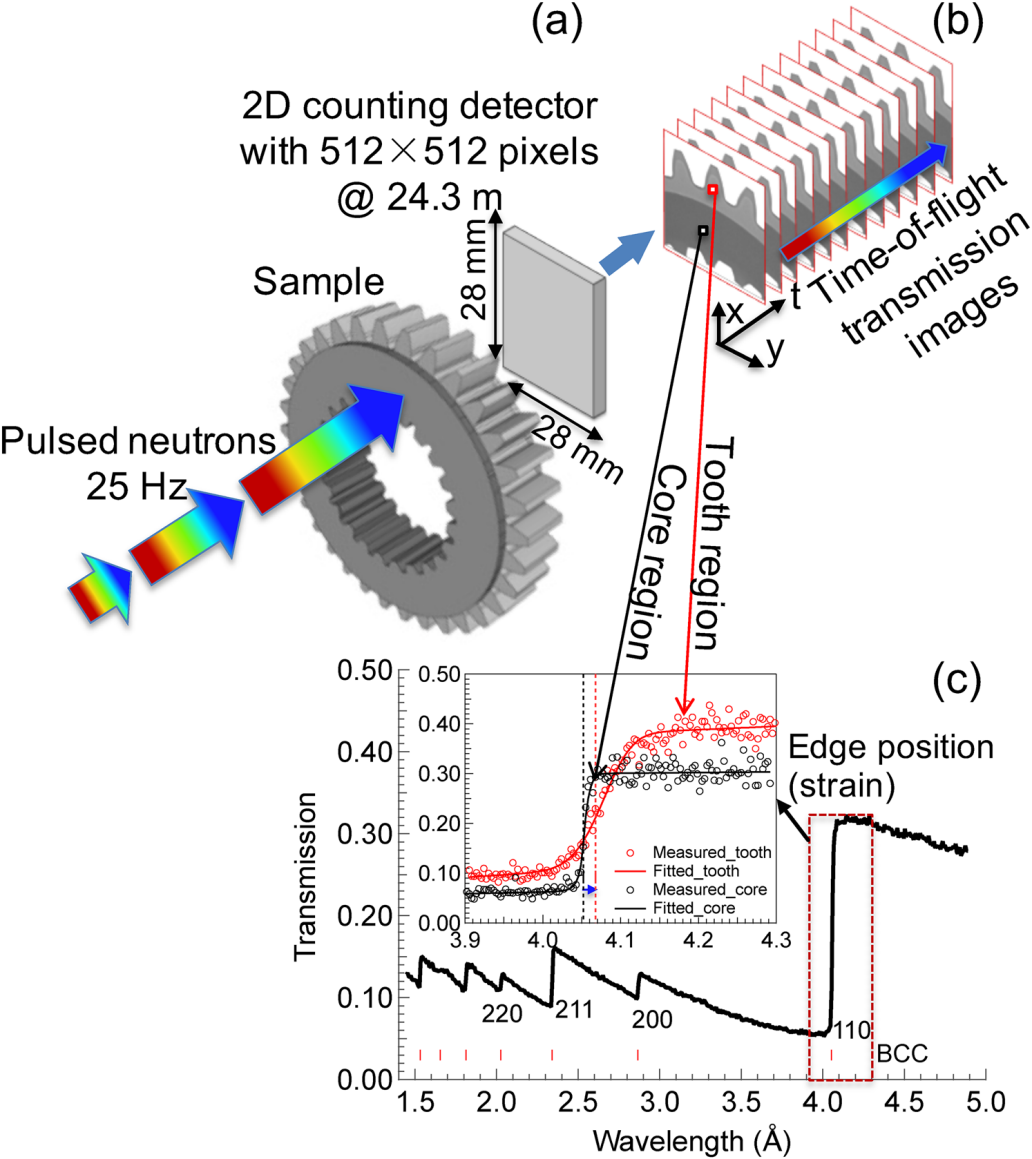
Schematic of the neutron Bragg-edge transmission imaging method: (**a**) Bragg-edge imaging experiment setup on the pulsed source; (**b**) the obtained energy-resolved neutron transmission images; and (**c**) an example of the Bragg-edge spectrum measured at the core region and BCC 110 single-edge profile fitting for edges from the core and the tooth region.

**Figure 2:**
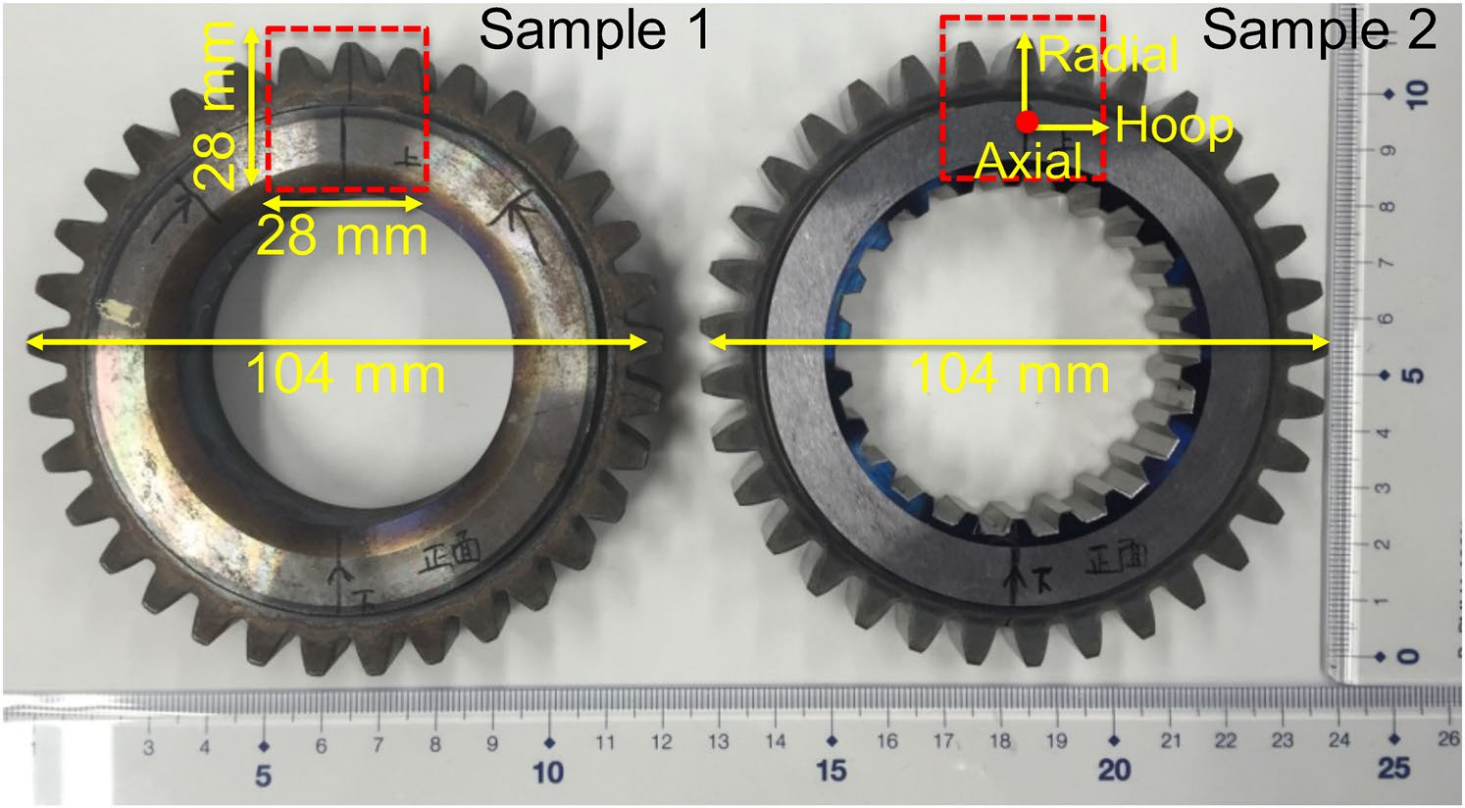
Photographs of the gears investigated in this work. The dashed boxes indicate the field of views of each sample examined by Bragg-edge imaging.

The Bragg-edge experiments were performed using the energy-resolved neutron imaging system, RADEN at MLF of J-PARC^24^ with 150 kW pulsed neutron beam operating at 25 Hz (Fig. 1). As shown in Fig. 1a, a 2D MCP (Microchannel Plates)/Timepix counting-type detector was positioned ~ 20 mm behind the gear sample. The detector has a high spatial resolution of ~ 55 µm and a total area of 28 × 28 mm^2^ with 512 × 512 pixels^25^. This allowed recording of the transmitted neutron intensity in the axial direction (through the thickness) of the measured gear samples as a function of TOF. The TOF was converted to the wavelength using the flight path length from the neutron source to the detector, which was determined to be 24.3 m using the standard iron sample data. The field of views for both samples is indicated by the dashed box in Fig. 2. Energy-resolved transmission images corresponding to wavelengths ranging from 1 to 6.5 Å were obtained (Fig. 1b) at a wavelength resolution of ~ 0.2% (Δλ/λ). As shown in Fig. 1c, a characteristic transmission spectrum, so-called Bragg edges, of the sample is measured by normalizing the neutron energy spectra with and without the samples. The RITS code was used for the Bragg-edge spectral analysis^26,27^. In this study, we used the body-centered cubic (BCC) ferrite 110 reflection for the single-edge analysis. The lattice plane spacing (Bragg-edge position), *d*_110_, and the Bragg-edge broadening, *w*_110_, were obtained via single-edge fitting^20^. 2D maps of *d*_110_, *w*_110_, and the residual strain of each sample were obtained by binning neutron counts of neighboring pixels over 3 × 3 pixels (~ 0.165 × 0.165 mm^2^).

The investigated gears (see Table 1 for the chemical composition) were manufactured from S55C steel, in accordance with JIS G4051 standard, by Neturen Co., Ltd. using the newly developed induction hardening technique. Figure 2 shows a photograph of the measured gear samples, namely Sample 1 and Sample 2, that were subjected to heat treatment procedure SIQ (single induction quenching) and DIQ, respectively. The DIQ process is a two-time induction hardening method combining standard induction hardening, SIQ, and contour hardening technology using super rapid induction heating and quenching (SRIQ)^22,23^. These two gears have 33 teeth, 20 mm and 15 mm thick in the gear body and tooth region, respectively, with an outer diameter of 104 mm and inner diameter of 52 mm. Sample 1, a precursor of the finished product, was produced using a standard induction hardening process, SIQ, to obtain a thick hardened martensite layer on the outer side of the gear. Sample 2, a finished product, was firstly subjected to the same induction hardening conditions (SIQ) as that of Sample 1. Afterward, the sample was preheated to obtain a tempered thermal refined microstructure and then subjected to a second induction hardening process, SRIQ. The SRIQ process generated very fine martensite and a large residual stress in the thin surface layer. As a result, the DIQ process led to the formation of a double-hardened layer (fine-grained martensite and tempered martensite) on the surface of Sample 2. Consequently, a large compressive stress, which helps to inhibit crack initiation and propagation, as well as improves the surface durability and fatigue strength, was expected at the surface of the DIQ gear.

**Table 1:** Chemical composition (wt%) of a steel for gears used in this study.

C	Si	Mn	P	S	Ni	Cr	Cu	Al
0.57	0.21	0.77	0.013	0.021	0.01	0.18	0.01	0.038

After the Bragg-edge imaging experiments, small pieces were cut from the samples and then polished and etched for macro- and microstructural analyses by means of optical microscopy. The XRD method was applied to measure the surface residual stress of each sample. The XRD stress measurements were performed using X-ray diffractometer with Cr-Kα radiation and BCC ferrite 211 reflection plane. The sin^2^*ψ* method was used to determine the residual stress. The incident radiation area is 0.5 mm in diameter controlled by using a collimator^28^. The hardness was also measured with a micro-Vickers hardness tester on the half-thickness of the gear. The depth profiles of hardness were obtained in steps of 0.05–0.1 mm in the hardened and transition zones and 0.2 mm in the core zone.

## Results and discussion

### Macro- and microstructural results

**Figure 3:**
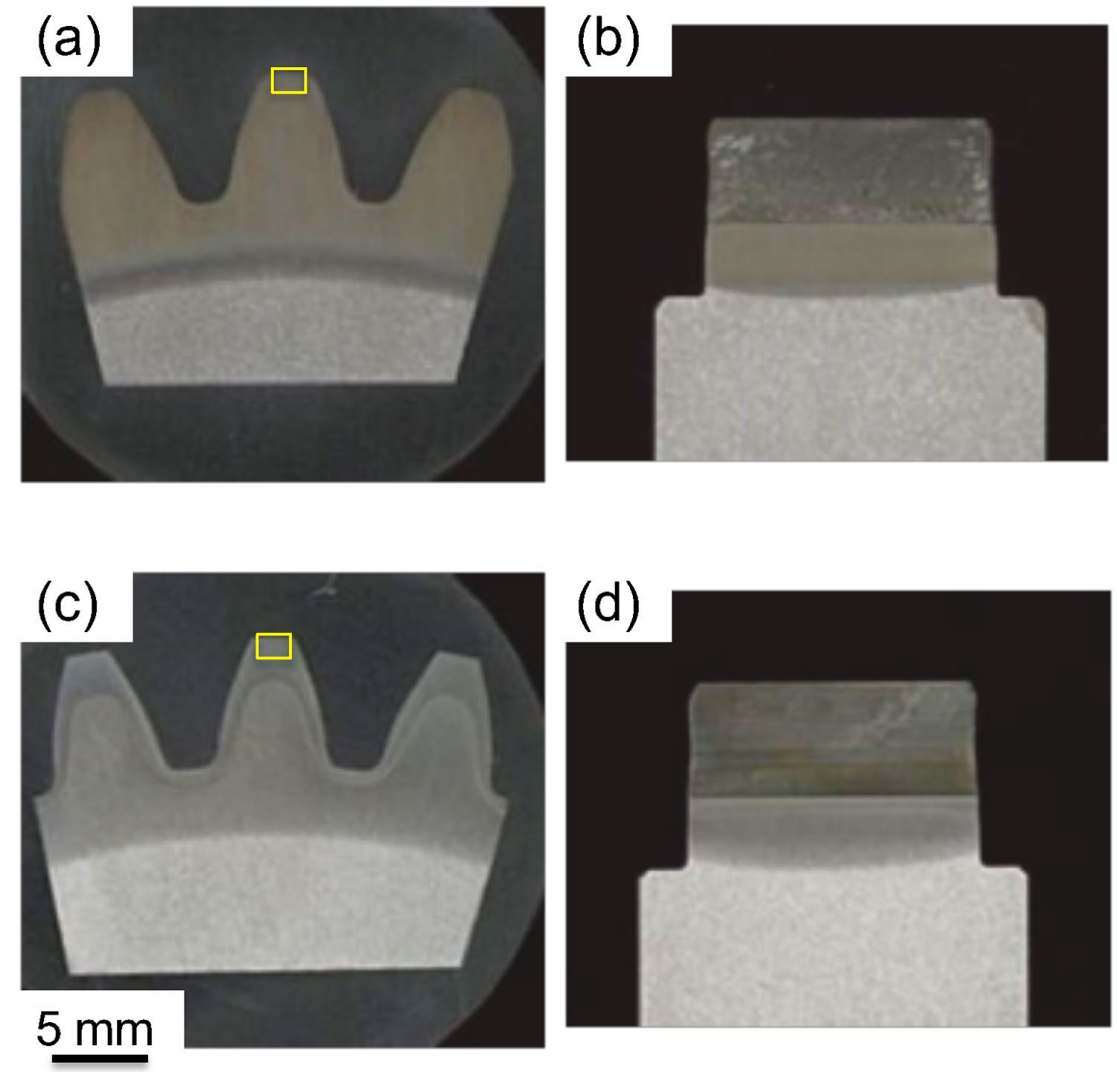
Optical micrographs showing the macrostructure of the tested gears: Sample 1 (**a**,**b**) and Sample 2 (**c**,**d**). (**a**,**c**) Obtained at the half gear thickness section observed from the axial (neutron transmission) direction and (**b**,**d**) were obtained for the gear tooth root plane observed from the hoop direction. The boxes in (**a**,**c**) indicate microstructure observation positions of each sample zooming in Fig. 5.

**Figure 4:**
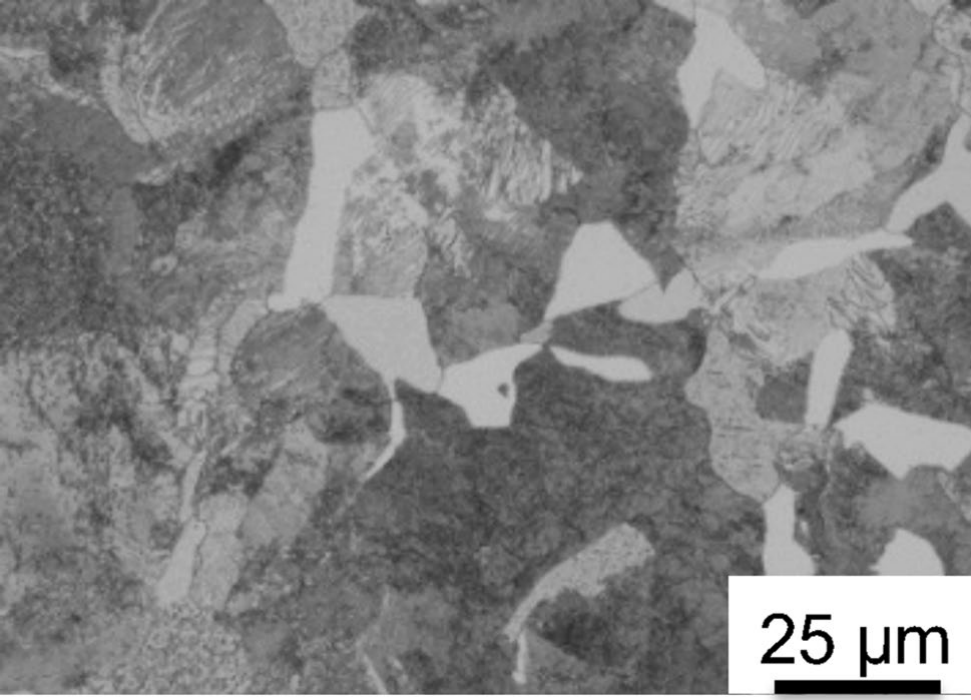
Initial microstructure prior to heat treatment of test gears, showing ferrite in grey color and pearlite in black color.

**Figure 5:**
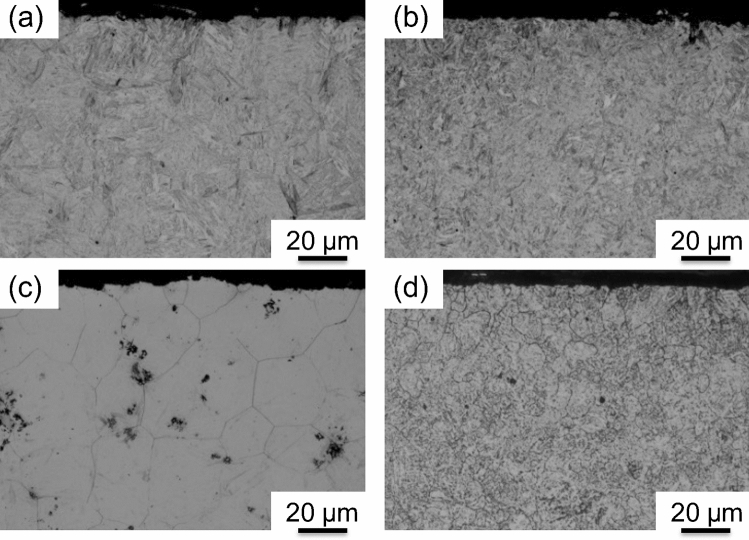
Optical micrographs of microstructures (**a,b**) and crystal grain (**c,d**) of test gears measured at the tooth tip of Sample 1 (**a,c**) and Sample 2 (**b,d**). The microstructure of martensite (**a,b**) was revealed by means of optical microscopy (OM). The crystal grain size (**c**,**d**) was determined by OM image analysis of the specimens according to ASTM-E112. The average grain diameter of Sample 1 and Sample 2 were estimated to be approximately 26.7 μm (ASTM grain size number #7.5) and 11.2 μm (#10), respectively. In addition, the dark spots in **c** were corrosion pits induced by inclusions during etching, which was performed strongly to reveal prior austenite grain boundaries.

Four-low magnification optical micrographs (macrographs) obtained at the half-thickness section from the axial direction and those of the tooth root plane from the hoop direction are shown in Fig. 3. A comparison of Fig. 3a,c revealed that different hardening patterns occurred in the two samples, i.e., a homogeneous hardening layer of the entire tooth and root was observed for Sample 1 (Fig. 3a), while a double-hardened layer occurred in Sample 2 (Fig. 3c). Macrographs in the tooth root plane of both samples (Fig. 3b,d) revealed that the hardening depth at the half-thickness position is greater than that at the near-surface position. This resulted from the fact that the non-uniform microstructural distribution was caused by the temperature gradients from the surface to the core of the sample during rapid induction heating. Interestingly, the positional shapes occurring in the core zone boundary of the root surface macrograph obtained for both samples were quite similar. This suggested that the microstructural changes at this boundary were induced by the SIQ process rather than the DIQ process. Figure 4 shows that the initial microstructure of the gear specimens prior to induction heat treating was composed of ferrite and pearlite phases. Microstructures at identical section of the tooth tip region in the half-thickness along the transmission (axial) direction of each sample after hardening were characterized by means of optical microscopy. As in the optical micrographs obtained at a high magnification (see Fig. 5a,b), the microstructure near the tooth tip of each sample was characterized by a fully martensitic structure. A comparison of Fig. 5c,d revealed that grain size of Sample 2 is obviously smaller than that of Sample 1.

### XRD results

The residual stress components in the hoop and axial (neutron transmission) directions at surface of tooth root for the two samples were determined by XRD measurement as tabulated in Table 2. The results show compressive residual stresses on the surface of both samples. Also, it can be seen that higher compressive residual stress exists in Sample 2 than that of Sample 1, which must be introduced by the DIQ treatment.

**Table 2:** Residual stresses near the surface of the tooth root of both gear samples determined by the conventional surface removal XRD method.

	Residual stress (MPa)	
	Axial	Hoop
Sample 1	− 189	− 356
Sample 2	− 515	− 1060

### Microhardness results

**Figure 6:**
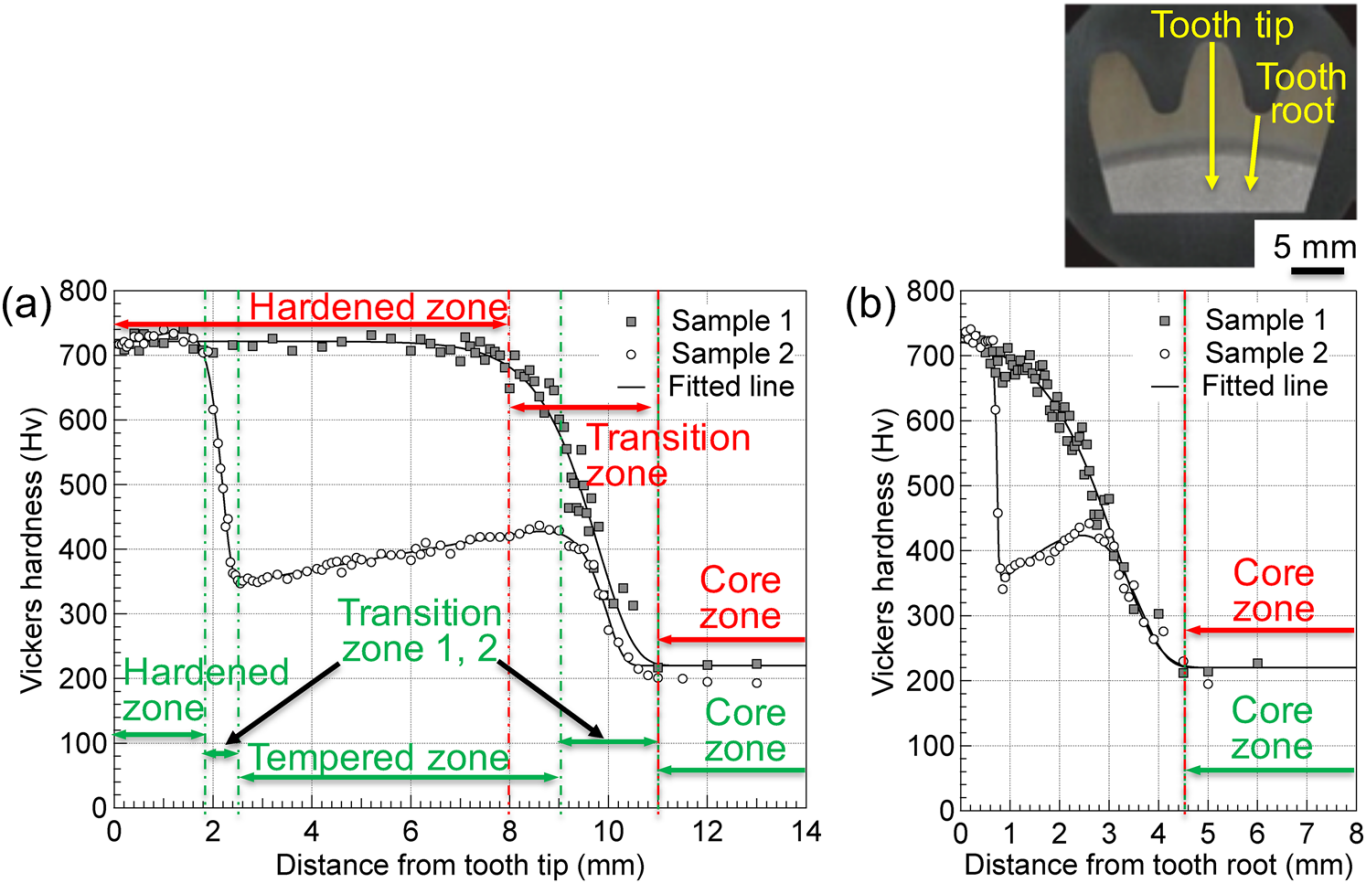
Hardness distribution profiles measured from the tooth tip (**a**) and the tooth root (**b**) for both gear samples.

As described in “Experimental”, Samples 1 and 2 were produced via SIQ and DIQ processes, which yielded a thick single martensite layer and a double martensite layer consisting of fine-grained martensite and tempered martensite. Microhardness profiles were measured from the tooth tip (Fig. 6a) and the tooth root (Fig. 6b) to the core of each sample, as indicated by the arrows in the cross-sectional view (square markers: Sample 1 and circle markers: Sample 2). Additionally, the results were fitted using a sigmoid-based temporary function (see the solid lines in Fig. 6). The Vickers hardness profiles shown in Fig. 6a indicated that Sample 1 consists of a hardened zone (0–8 mm), transition zone (8–11 mm), and core zone (> 11 mm) spanning the tooth tip to the core region. The hardness was almost uniform in the hardened zone, where the hardness value Hv was slightly  higher than 700. This value resulted from martensite formation induced by induction quenching. In the transition zone, Hv decreased gradually to a minimum value of ~ 200. The transition zone was considered a region with a mixed microstructure of martensite and ferrite, or martensite, ferrite, and pearlite. In the core zone, the Hv remained at a nearly constant value of slightly greater than 200, which is the typical hardness of unheated S55C steel consisting of ferrite and pearlite phases. The SIQ process had no effect on the microstructure in this zone. However, five distinct zones occurred from the tooth tip to the core region of Sample 2, which was subjected to the DIQ process, i.e., a hardened zone (0–1.8 mm), transition zone 1 (1.8–2.5 mm), tempered zone (2.5–9 mm), transition zone 2 (9–11 mm), and core zone (> 11 mm). Additionally, the slope of the hardness profile obtained for transition zone 1 was considerably steeper than that of transition zone 2. This was indicative of the subsurface microstructural variations resulting from different temperature distributions associated with the heat treatment. The hardness of the hardened zone near the tooth tip was also uniform with values near 700. However, a shallower hardening depth than that of Sample 1 (resulting from the second hardening process, SRIQ, after the quenching followed by a tempering process, referred to as thermal refining) was indicated. The hardness of the tempered martensite zone (minimum Hv: ~ 360) increased slowly to a maximum of ~ 430, owing to the thermal flow occurring during the induction hardening process, which influences the assumed temperature gradients and may have effects on the microstructural evolution that yields the slowly increasing hardness in this zone. The hardness then decreased gradually to a minimum value of 200 in the core zone, similar to the trend observed for Sample 1. The microstructure in transition zone 1 consisted of quenched and tempered martensite, while the microstructure in transition zone 2 consisted of tempered martensite and a core structure of ferrite and pearlite. The single-hardened layer due to the SIQ treatment and double-hardened layers induced by the DIQ treatment were observed from the hardness profiles of Sample 1 and Sample 2, respectively. Similarly, the hardness distribution profiles from the tooth root to the core shown in Fig. 6b corresponded to the single layer of Sample 1 and the double-hardened layers of Sample 2. In Sample 1, the boundary between the hardened and transition zones was barely visible. In contrast, in Sample 2, the five zones with Hv values similar to those of the tooth tip and their boundaries were clearly visible. The hardened zone occurring in the tooth root of this sample was much shallower than that of the tooth tip, although, the Hv value and the microstructure were almost the same as those of the tip.

### Bragg-edge imaging results

**Figure 7:**
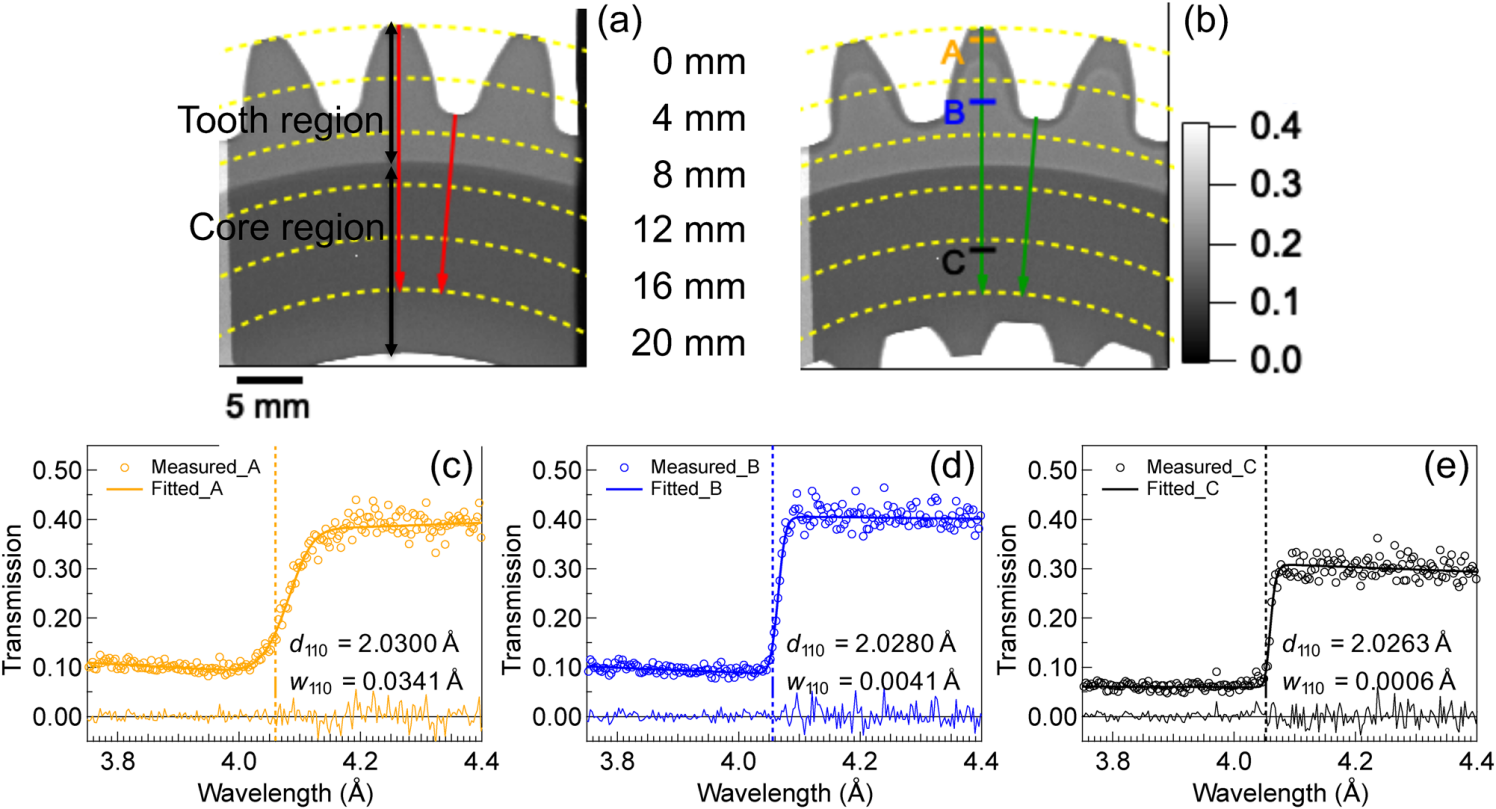
Neutron Bragg-edge transmission images obtained at wavelengths of 3.46–4.51 Å around the 110 edge: (**a**) for Sample 1 and (**b**) for Sample 2. Representative transmission spectra of gears obtained at point A of the hardened zone (**c**), point B of the tempered zone (**d**), and point C of the core (**e**) for Sample 2. The dashed line indicates the edge positions given by the refinement. The plot at the bottom indicates the difference.

Figure 7a,b show neutron transmission images of Sample 1 and Sample 2 accumulated over wavelengths ranging from 3.46 to 4.51 Å across the BCC 110 edge measured by the MCP detector. Concentric dashed partial circles represent the distances from the tooth tip to the center of the gear of 0, 4, 8, 12, 16, and 20 mm. The gear thickness in the region corresponding to distances of 0 to 10 mm from the tooth tip was 15 mm, and the thickness of the rest of the gear was 20 mm. In this paper, these regions are referred to as the tooth region and the core region. The transmission in the tooth region of each sample was significantly higher than that of the core region, owing to the lower thickness in this region. The transmission in the core region of each sample was uniform and similar, suggesting that the same matrix microstructure occurred in both samples. However, the energy-resolved transmission image for the tooth region of each gear revealed different contrasts despite the same shape and thickness of the gears. These contrasts arise from differences in the microstructure and the resulting accumulated transmission value. Results shown in Fig. 7c–e represent examples of the single-edge profile fitting for the 110 lattice plane at the hardened zone, tempered zone, and core zone of Sample 2 with size of 30 × 3 pixels (1.65 × 0.165 mm^2^): position A, position B, and position C located at ~ 1 mm, ~ 5.8 mm, and 17 mm, respectively, from the tooth tip (see Fig. 7b). Refined 110 lattice plane spacing values, *d*_110_, of 2.0300 Å, and 2.0263 Å, which are basically consistent with those of the martensite and ferrite phases, were determined for positions A and C, respectively. Additionally, Bragg-edge broadening was confirmed at positions A and B. This indicated the formation of a martensitic microstructure in the hardened zone and tempered zone due to induction heating.

**Figure 8:**
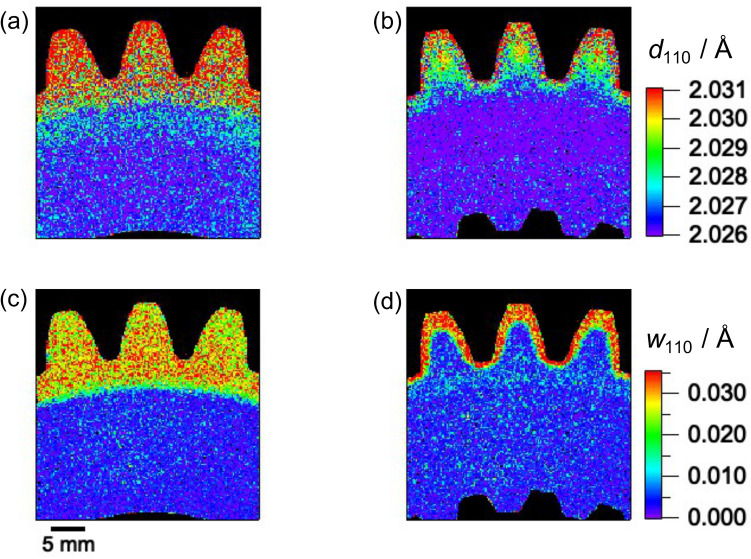
2D maps obtained via Bragg-edge imaging: (**a**,**b**) 110 lattice plane spacing, *d*_110_ and (**c**,**d**) the Bragg-edge broadening of the 110-crystal lattice plane spacing, *w*_110_, where (**a**,**c**) are for Sample 1 and (**b**,**d**) for Sample 2. The pixel size is ~ 0.165 mm^2^.

Figure 8 shows 2D maps of the obtained lattice plane spacing, *d*_110_, and the Bragg-edge broadening, *w*_110_. Two kinds of regions, the hardened zone and the core zone, were clearly observed in the *d*_110_ map of Sample 1 (Fig. 8a). This indicated the formation of a martensite structure with lattice spacing of ~ 2.031 Å in the tooth hardened region and a ferrite matrix in the core with a value of ~ 2.026 Å. In addition to the martensite hardened zone similar to that of Sample 1, a tempered region was visualized in the *d*_110_ map of Sample 2 (green region in Fig. 8b). The single layer of quenched martensite in Sample 1 and double martensite layers in Sample 2 were recognized from the *w*_110_ maps shown in Fig. 8c,d, respectively. The map of *w*_110_ was similar (in general) to the map of *d*_110_ and the boundaries between the zones in the former were more visible than those in the latter. However, the correlation between *d*_110_ and *w*_110_ did not seem to be linear. Additionally, the *w*_110_ values corresponding to the near-surface layer of Sample 2 (Fig. 8d) seemed to be larger than those of Sample 1 (Fig. 8c). This may have resulted from the smaller martensite grain size of Sample 2 (compared with that of Sample 1), as suggested by the microstructural observation results shown in Fig. 5b,d. Therefore, non-contact and non-destructive 2D microstructural mapping of the induction hardened gear can be realized via the Bragg-edge imaging method.

**Figure 9:**
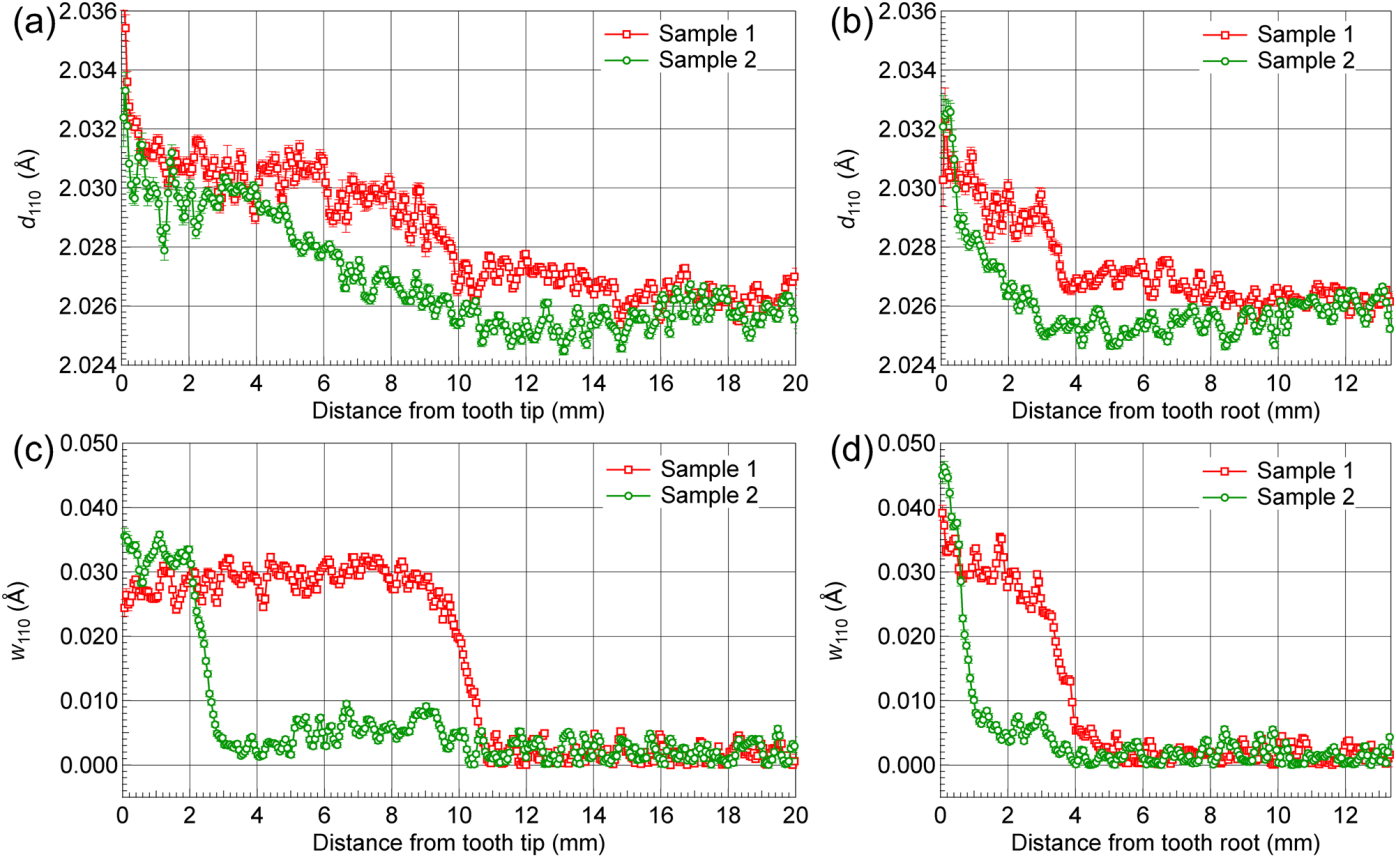
Changes in the lattice plane spacing, *d*_110_, (**a**,**b**) and the Bragg-edge broadening of the 110-crystal lattice plane spacing, *w*_110_, (**c**,**d**) for both test gears from tooth tip and root to core region, as indicated by the arrows in Fig. 7a,b.

The difference between the microstructural distributions in Sample 1 and Sample 2 was quantitatively investigated via analysis of transmission spectra obtained along the radial direction from the tooth tip to core and from the tooth root to the core. These are indicated by the arrows in Fig. 7a,b. To obtain sufficient counting statistics for the analysis, spectra for a 30 × 3 pixel area (1.65 mm in horizontal direction and 0.165 mm in vertical direction) were summed into a single spectrum. The area was moved at intervals of one pixel in vertical direction. Figure 9 presents the obtained lattice spacing, *d*_110_, and the Bragg-edge broadening, *w*_110_, distribution profiles as a function of distance from the tooth tip (Fig. 9a,c) and the tooth root (Fig. 9b,d) of each sample. As shown in Fig. 9a, the *d*_110_ of Sample 1 was almost constant from the tooth tip to a depth of 6 mm. Subsequently, this value decreased in an arch-like manner toward the core until the depth reached ~ 10 mm, and then decreased very slowly from 10 to 15 mm. Finally, a nearly constant value of ~ 2.026 Å was reached from ~ 15 mm, revealing the boundary of the unaffected core zone. This indicated that the quenched martensite layer extended to ~ 6 mm from the surface, which is roughly consistent with the hardness distribution results of Sample 1 (see Fig. 6a). However, the subsequent transition layer (ranging from 6 to 15 mm) was considerably wider than that observed from the Hv profile (8–11 mm) of the sample (Fig. 6a). A very slow decrease region occurred at depths of 10–15 mm of the sample. This may have resulted from the inhomogeneous microstructural features such as carbon concentration distribution in martensite or residual stress generation during the SIQ hardening process. However, a different trend was observed for the *d*_110_ distribution profile of Sample 2, i.e., *d*_110_ decreased gradually from the tooth tip and reached a constant value of ~ 2.026 Å upon approaching a distance of 15 mm, suggesting that the core material zone with ferrite and pearlite matrix was reached. Distinguishing the hardened zone and tempered zone based on this profile was difficult. This may have resulted from the complex microstructure including fine martensite, tempered martensite, ferrite/pearlite, or the presence of carbon concentration/residual stress distribution due to the DIQ treatment. As shown in Fig. 9c, the overall *w*_110_ profiles for both samples are similar to those of microhardness profiles shown in Fig. 6a. Three zones were observed for Sample 1: the hardened zone, transition zone, and core zone, indicative of microstructural transitions from the tooth tip to the core. Similarly, the *w*_110_ profiles of Sample 2 indicated five zones: the hardened martensite zone, tempered martensite zone, core zone from the tooth tip to the core, and two transition zones between these regions. Moreover, similar trends were observed for the *d*_110_ and *w*_110_ profiles obtained from the tooth tip and the tooth root to the core of both samples (see Fig. 9b,d). The *w*_110_ profiles shown in Fig. 9d are roughly consistent with the Hv profile shown in Fig. 6b (core depth at the tooth root of each sample: ~ 4.5 mm). Additionally, the value of *w*_110_ in the outermost part of Sample 2 was substantially larger than that of Sample 1, as shown in Fig. 9c,d, as well as in Fig. 8c,d. These Bragg-edge analyses successfully visualized the differences in the transition zone band widths and hardening depths of both samples in two dimensions. In the following section, we investigate the relationship between the Vickers hardness and *w*_110_ of each sample.

### Comparisons of the Bragg-edge broadening and microhardness

**Figure 10:**
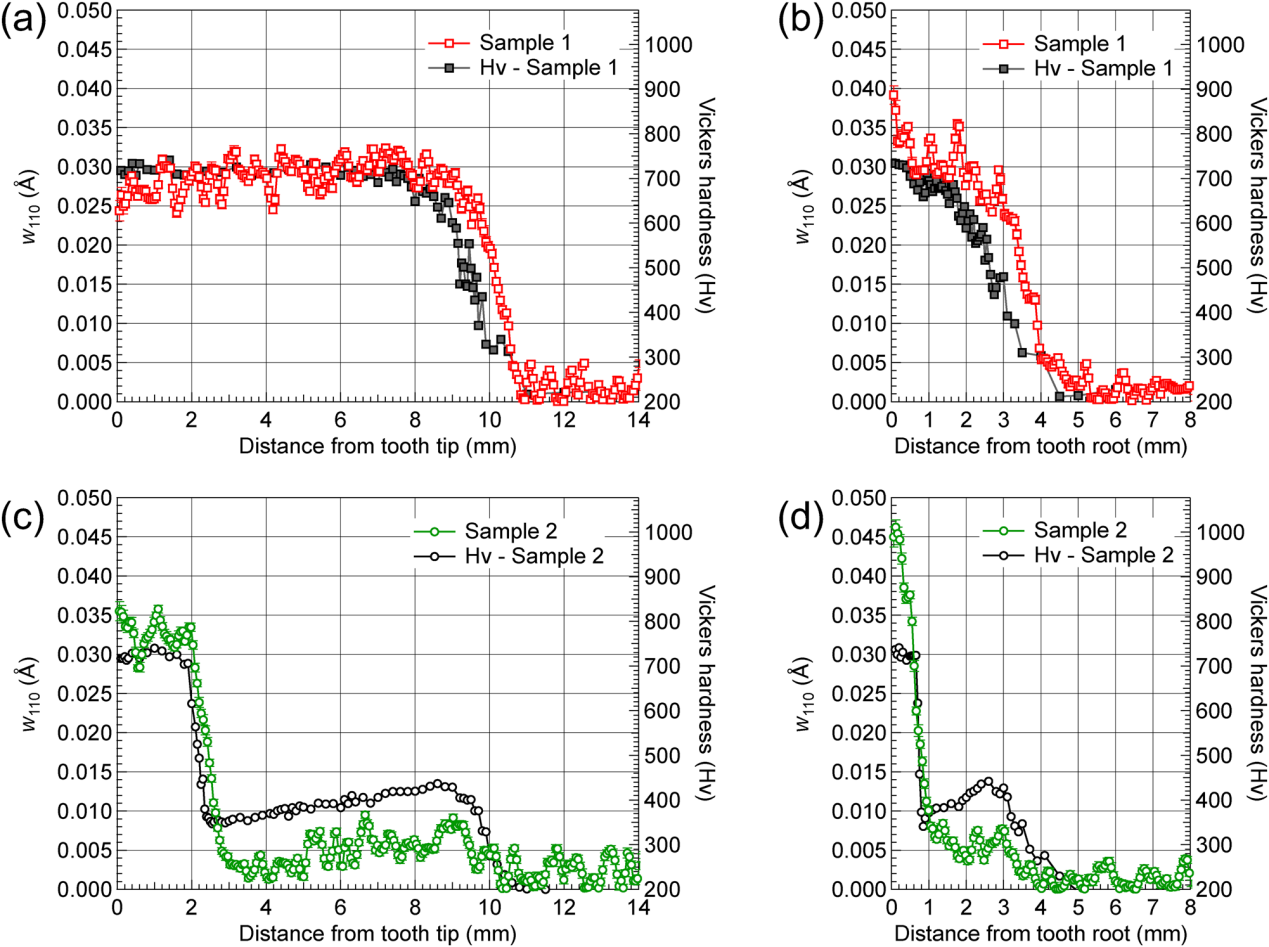
Changes in micro-Vickers hardness and the Bragg-edge broadening of the 110-crystal lattice plane spacing, *w*_110_, plotted against distance from the tooth tip of each gear (**a**,**c**) and tooth root (**b**,**d**), as indicated by the arrows in Fig. 7a,b.

The present data analysis revealed that the position dependence of the Bragg-edge broadening, *w*_110_, is similar to that of the Vickers hardness, Hv, as determined by Sato et al., who investigated simple rod-shaped quenched steels^26^. The Hv fitting result for Sample 1 (Fig. 6a) revealed average Hv values of 720 (average *w*_110_: 0.03 Å) and 220 (average *w*_110_: 0.002 Å; see Fig. 9c) for the hardened and core zones, respectively, from which a simplified linear relationship Hv = 184 + 17,857 *w*_110_ is derived. Figure 10 shows the obtained *w*_110_ and the measured Hv as a function of distance from the tooth tip and the tooth root of both samples, scaled by this relationship. As shown in Fig. 10a,b, the relative changes in the *w*_110_ profile are similar to those of the Hv profile. However, the slope positions of *w*_110_ and Hv profiles in the transition zone are slightly different. The trends of *w*_110_ and Hv for Sample 2 (Fig. 10c,d) are, however, somewhat different, especially in the transition and tempered zones. Moreover, only part of the *w*_110_ profile can be described by a linear relationship with Hv, unlike the simple system reported by Sato et al., who considered quenched steel rods. The non-uniform carbon distribution is caused by the phase transformation occurring during the induction heat treatment process with different temperature gradients^29,30^. For the induction hardened gears, the microstructure/carbon content of the samples varies from the tooth tip/root, which may include (1) the martensite (transformed from austenite single-phase during quenching) region near the surface, followed by (2) the martensite-ferrite two-phase structure transition region, then (3) the martensite-pearlite-ferrite transition region, and (4) the unaffected pearlite-ferrite base region. The carbon content of martensite in the transition regions (2) and (3) would be higher than that of region (1). Several factors (e.g., non-uniform carbon content, dislocation density, grain shape/size, and carbide precipitates) have differing effects on the *w*_110_ and Hv^31,32^. Then, the *w*_110_ and Hv increased (in general) with increasing volume fraction of martensite. Generally, in most materials, the Vickers hardness is roughly three times of the yield strength^33^. In fact, as reviewed by Tomota et al.^34^, the yield strength (i.e., hardness) deviates below the line predicted from the simple law of mixtures in the two-phase region of two-ductile-phase alloys, which is controlled by the soft phase. This may have resulted in the deviation of the predictions from the linear relationship between the *w*_110_ and Hv shown in Fig. 10, which revealed a linear relationship in the martensite hard phase region. The relationship was, however, invalid for the mixed-phase transition zone. However, in the following discussion, a model of microstructural changes described by a very simplified linear relationship (Hv = 184 + 17,857 *w*_110_) between *w*_110_ and Hv was assumed. This model was used to derive the elastic residual strain distribution of each sample*.*

### Residual strain distributions

The elastic residual strain distribution introduced into the martensite and transition phases during induction hardening is calculated from the following equation:

1 $$\varepsilon_{{{11}0}} = (d_{{{11}0}} - d_{0} )/d_{0}$$

where *d*_0_ is the stress-free reference lattice spacing for the 110 plane. To estimate the position dependent *d*_0_ values, we considered the stress-free 110 lattice spacing of ferrite (*d*_F_) and martensite (*d*_M_) of the samples. A *d*_F_ of 2.026 Å was determined from single-edge profile fitting applied to the transmission spectra of the core region. A *d*_M_ of 2.0385 Å was deduced from the published relations between the lattice parameters and the interstitial carbon content of iron-carbon martensite^35^ (a carbon content of 0.57 was adopted). From *d*_M_ and *d*_F_, the stress-free lattice spacing of 110 associated with the mixed phases in the transition zone (*d*_M+F_) is given by the following simple equation:

2 $$d_{{{\text{M}} + {\text{F}}}} = f \cdot d_{{\text{M}}} + (1 - f) \cdot d_{{\text{F}}}$$

where *f* is the martensite volume fraction.

A simple linear relationship between Hv and the obtained *w*_110_ is lacking, as described in “Microhardness results”. In general, the Hv of a two-phase alloy is lower than the value predicted by a simple linear relationship, i.e., a law of mixtures. Speich et al. have considered some DP steels with a soft ferrite matrix containing a hard martensitic phase^4^. Those authors have reported a linear proportional relationship between *f* and the Vickers hardness values of martensite (Hv_M_), ferrite (Hv_F_), and the mixed phase (Hv_M+F_), which is given as follows:

3 $$\left( {{\text{Hv}}_{{{\text{M}} + {\text{F}}}} } \right) = f \cdot {\text{Hv}}_{{\text{M}}} + ({1} - f) \cdot {\text{Hv}}_{{\text{F}}}$$

where the Hv_M_ and Hv_F_ values of our samples were estimated to be 720 and 220, respectively.

Moreover, to the best of our knowledge, a generalized correlation model between the Hv and *w*_110_ of multi-phase microstructures is lacking. The strain in this work was, therefore, assessed by assuming a model microstructural gradient expressed by a simple equation (e.g., Eqs. (2) and (3)).

**Figure 11:**
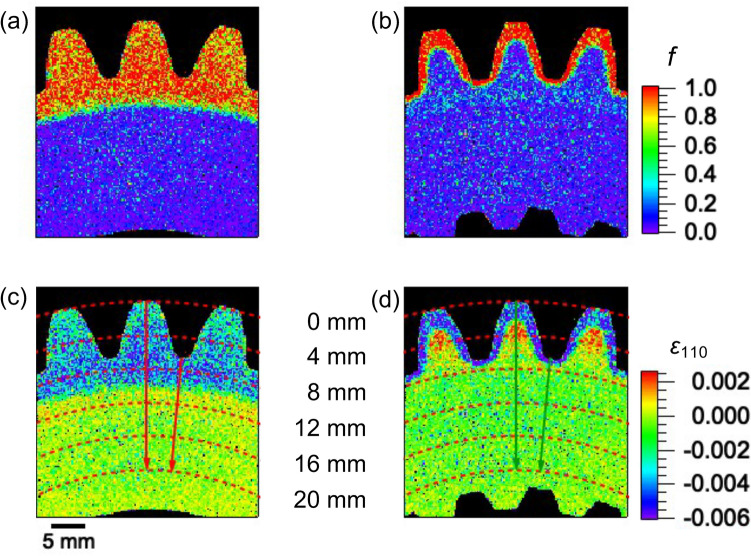
2D maps obtained via Bragg-edge imaging: (**a**,**b**) martensite volume fraction, *f*, and (**c**,**d**) residual strain of 110 lattice plane, *ε*_110_, where (**a**,**c**) are for Sample 1 and (**b**,**d**) for Sample 2. The pixel size is ~ 0.165 mm^2^.

As shown in Fig. 11a,b, the 2D maps of the martensite volume fraction, *f*, were obtained from Fig. 8c,d by replacing Hv with *w*_110_ in Eq. (3). The *f* maps indicate near 100% martensite in the hardened layer and 0% in the core, revealing the difference between the marteniste distributions of the two samples. The 2D maps of the stress-free lattice plane spacing of 110, namely *d*_M+F_, for the two samples were calculated by substituting *d*_M_, *d*_F_, and 2D map values of *f* into Eq. (2). Subsequently, the residual strain, *ε*_110_, of both samples was obtained (from Eq. (1)) and mapped in Fig. 11c,d. The residual strain distributions differed considerably between the two samples due to the different heat histories of the SIQ and DIQ processes. In Sample 1 (Fig. 11c), the SIQ process produced residual compressive strain extending inward from the outer surface and transitioning to a strain near zero. In contrast, in Sample 2 (Fig. 11d), the DIQ process produced compressive strain at the hardened martensite surface layer, tensile strain at the tempered martensite zone, and near-zero strain at the core region. Tensile strain values in the red-colored area around the center of the tooth (the tempered martensite zone) seemed large, because Hv was replaced with *w*_110_ in Eq. (3). These values may decrease if the Hv values are used.

**Figure 12:**
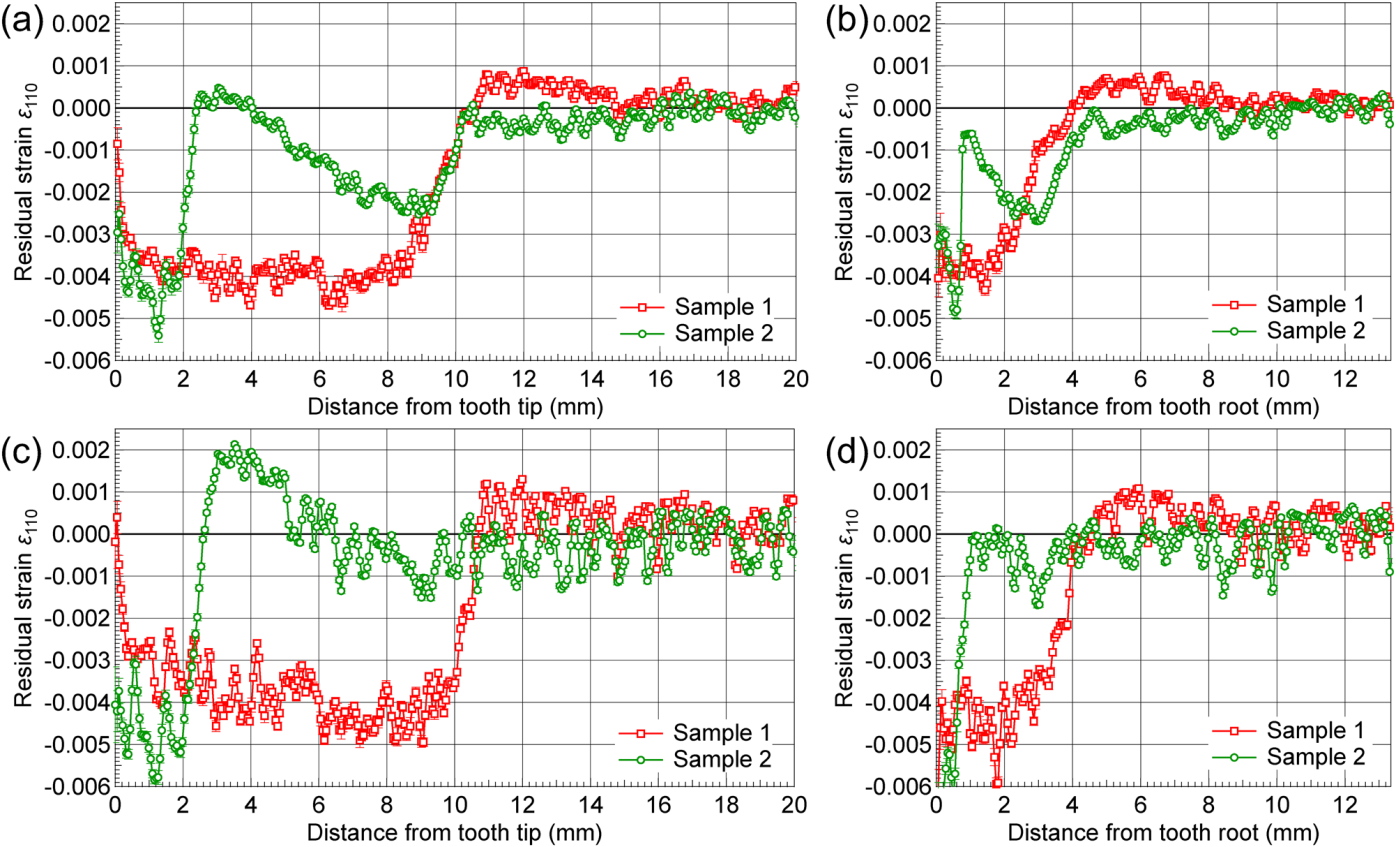
Residual strain of 110 lattice plane, *ε*_110_, determined via Bragg-edge imaging: from tooth tip (**a**,**c**) and tooth root (**b**,**d**) to core, as indicated by the arrows in Fig. 11c,d, where (**a**,**b**) are calculated from *d*_110_ values in Fig. 9a,b, and the fitted Hv values in Fig. 6a,b., and (**c**,**d**) are calculated from *d*_110_ values in Fig. 9a,b, and the *w*_110_ values in Fig. 9c,d.

To estimate the applicability of the obtained 2D residual strain maps, we calculated the residual strain profiles from the tooth tip/root to the core region along the radial direction using *d*_110_ and Hv values. Residual strains were calculated from Eqs. (1–3), from the *d*_110_ values in Fig. 9a,b, and the fitted Hv values in Fig. 6a,b. Strain profiles obtained from the tooth tip differed significantly between the two samples, as shown in Fig. 12a. In Sample 1, an almost constant value of ~  − 0.004 in the surface martensite hardened layer was observed up to a depth of 8 mm, which suggested that a large compressive residual strain was present. Moreover, as shown in Table 2, XRD analysis yielded a residual stress value of σ_Axial_ =  − 515 MPa and σ_Hoop_ =  − 1060 MPa at these surface areas. The compressive residual strain value was roughly estimated as − 0.000985, from Hooke’s law in plane stress:

4 $$\varepsilon_{{{\text{Axial}}}} = \left( {{1}/E} \right) \, (\sigma_{{{\text{Axial}}}} - \nu \cdot \sigma_{{{\text{Hoop}}}} )$$

assuming that Young’s modulus *E* = 200 GPa and Poisson’s ratio *v* = 0.3. The absolute values of the residual strain distributions differed between the results of the Bragg-edge imaging and XRD, which are reflected in the uncertainty in the *d*_0_ values used. However, the rough estimation is consistent with the reported XRD-determined residual stress values (on the order of − 1000 MPa) measured at surface regions. The compressive residual strain decreased gradually and became a weak tensile strain at distances ranging from 11 to 15 mm, approaching zero at ~ 15 mm, corresponding to the unaffected core zone boundary of Sample 1. In Sample 2, a compressive residual strain at the hardened surface layer increased sharply and became a tensile strain at ~ 2 mm. Afterward, the compressive strain increased again in the tempered zone from 3 to 9 mm and decreased to 0 at ~ 12 mm. Moreover, the compressive residual strain in the hardened area induced by the DIQ treatment of Sample 2 is larger than that in Sample 1, which is consistent with the XRD results. As reported in previous studies^1,22,23^, the compressive strain at the surface, balanced by a tensile strain at the interior of the component will result in improved fatigue performance of the gear. The residual strain distribution of Sample 2 resulting from the DIQ process must be favorable for the bending fatigue resistance strength. Residual strain profiles obtained for regions ranging from the tooth root to the core (see Fig. 12b) exhibited similar trends to those of the profiles obtained for the tooth tip. A compressive strain occurred to a depth of ~ 4 mm in both samples. Moreover, the profiles of residual strain were obtained using the same procedure described in the previous paragraph. Strains were calculated from Eqs. (1–3), from *d*_110_ values in Fig. 9a,b, and the *w*_110_ values in Fig. 9c,d. As shown in Fig. 12c,d, the variation tendency of the residual strain distributions for both samples is similar to those shown in Fig. 12a,b. Strain values in the transition and tempered martensite regions were, however, somewhat shifted in the positive direction, as observed in the values of the tempered martensite region of the 2D map shown in Fig. 11d. The shift resulted mainly from the low-*f* values in this region, which stemmed possibly from the different influential factors for *w*_110_ and Hv in the tempered martensite. That is, Hv increased with increasing volume fraction of carbides, whereas *w*_110_ changed only slightly, as described in “Microhardness results”.

Therefore, the residual strain distributions obtained from Bragg-edge imaging are qualitatively consistent with the results of XRD, although inaccuracies occur in the strain values calculated by the simplified method. Issues persist regarding the accurate estimation of the *d*_0_ values. However, the 2D distributions of microstructure and elastic residual strain in two induction hardened samples are non-destructively visualized for the first time with the use of the TOF Bragg-edge imaging analysis. This technique would be applicable to other induction-hardened products or heat-treated machine parts with two-dimensional structural features such as the plane strain state. From the viewpoint of transmission ability, the measurement for steel samples with the thickness approximately up to 20 mm is possible. In addition, three-dimensional residual strain experiment for the development of strain tomography techniques has started at RADEN that could be applied to complex engineering components in the future^36,37^.

## Conclusions

In this work, the microstructural features associated with two kinds of induction hardened gears were non-destructively evaluated by a neutron Bragg-edge imaging method. The microstructure and strain distributions of two gear samples, Sample 1 subjected to the SIQ process and Sample 2 subjected to the DIQ process, have been successfully visualized. The results of this work are summarized as follows.

- The 2D maps of the lattice plane spacing, *d*_110_, and Bragg-edge broadening, *w*_110_, were obtained via Bragg-edge imaging with high spatial resolution. The single layer of quenched martensite and double-hardened layers consisting of fine-grained martensite and tempered martensite were observed in Sample 1 and Sample 2, respectively.
- The variation in the determined Bragg-edge broadening, *w*_110_, from the tip/root of the gear teeth region to the core region was similar to that of the Vickers hardness, Hv. However, the formulation of a simple linear relation for describing the relationship between the *w*_110_ and Hv was difficult due to the phase transformation with carbon diffusion induced by induction hardening.
- The 2D maps obtained for the elastic residual strain, *ε*_110_, of the two gear samples represent the first-ever maps of this type obtained using the Bragg-edge imaging method. Compressive residual strains were generated in the martensite hardened zone of both gears, while near-zero residual strains were generated in the core. Particularly, a steep compressive residual strain in the hardened layer induced by the DIQ treatment of Sample 2 was confirmed, which agrees with the XRD measurement results. Although the *d*_0_ estimation method needs improvement, the Bragg-edge analysis method could be a powerful non-destructive method for tracking the microstructural and residual strain evolutions in the hardened gears.
